# *Recticladiella inexpectata* gen. et sp. nov. (Nectriaceae) Pathogenic to Native *Cinnamomum camphora* (Lauraceae) Trees in Southeastern China

**DOI:** 10.3390/jof10120894

**Published:** 2024-12-23

**Authors:** Fangying Han, Shuaifei Chen

**Affiliations:** Forest Pathogen Center (FPC), College of Forestry, Fujian Agricultural and Forestry University, Fuzhou 350002, China

**Keywords:** *Eucalyptus*, Hypocreales, native tree, inoculation tests, phylogeny, tree pathogens

## Abstract

The ascomycete family Nectriaceae includes soil-borne saprobes, plant pathogens and human pathogens, biodegraders, and biocontrol agents for industrial and commercial applications. *Cinnamomum camphora* is a native tree species that is widely planted in southern China for landscaping purposes. During a routine survey of *Eucalyptus* diseases in southern China, disease spots were frequently observed on the leaves of *Ci. camphora* trees planted close to *Eucalyptus*. The asexual fungal structures on the leaf spots presented morphological characteristics typical of the Nectriaceae. The aim of this study is to identify these fungi and determine their pathogenic effect on *Ci. camphora*. Of the isolates obtained from 13 sites in the Fujian and Guangdong Provinces, 54 isolates were identified based on the DNA phylogeny of the *tef1*, *tub2*, *cmdA*, and *his3* regions and morphological features. Two isolates were identified as *Calonectria crousiana*, and fifty-two isolates were described as a new genus, including a single species. These fungi were named *Recticladiella inexpectata* gen. et sp. nov. The identification of the new genus was based on strong DNA base differences in each of the four sequenced gene regions. The conidiophores of this fungus had several avesiculate stipe extensions tapering toward a straight, occasionally slightly curved terminal cell, distinguishing it from other phylogenetically close Nectriaceae genera. The results indicate that *R*. *inexpectata* is distributed in wide geographic regions in southern China. Inoculation showed that *R*. *inexpectata* and Ca. *crousiana* caused lesions on the leaves of *Ci. camphora* seedlings within 6 days of inoculation, indicating that they are pathogenic to native *Ci. camphora* in China.

## 1. Introduction

The ascomycete family Nectriaceae Tul. and C. Tul. (Hypocreales Lindau, Hypocreomycetidae O.E. Erikss. and Winka, Sordariomycetes O.E. Erikss. and Winka, and Pezizomycotina O.E. Erikss. and Winka) is characterized by uniloculate ascomata that are white, yellow, and orange-red to purple and associated with phialidic asexual morphs producing amerosporous to phragmosporous conidia [1,2]. Members of the Nectriaceae are found in various environments, and some species are important human or plant pathogens [3]. Lombard et al. [3] resolved most taxonomic discordances within the family of Nectriaceae based on morphological and molecular phylogenetic analyses, and 47 genera were re-evaluated. Based on the most recent results, at least 79 genera within this family were accepted [4,5,6]. The Nectriaceae includes numerous important plant pathogens, such as *Fusarium* Link and *Calonectria* De Not. species [7,8,9]. These pathogens are causal agents of fruit rot, leaf blight, leaf spot, stem canker, branch wilt, and root rot in many forestry, agricultural, and horticultural plants across the globe [3,10,11].

*Cinnamomum camphora* (L.) J. Presl (Lauraceae Juss, Laurales Juss. ex Bercht. and J. Presl) is an evergreen tree species that is widely distributed in subtropical regions, including China, Japan, and northeastern Australia [12,13]. *Cinnamomum camphora* is native to China and distributed in approximately 14 provinces (autonomous regions), especially in southern, southeastern, and southwestern regions of the country [14,15]. In China, *Ci. camphora* trees are mainly used for landscaping purposes [16] and as furniture and building materials [17]. *Cinnamomum camphora* tree oil extracted from these trees is widely used to produce medicine, cosmetics, pesticides, and repellents [18,19].

Research on Nectriaceae fungi in China began in 1982, and the first specimen of this group of fungi was collected in Hangzhou, Zhejiang Province. The fungus *Calonectria uredinophila* Syd. and P. Syd. was identified as a novel species and recently renamed *Nectriopsis uredinophila* (Syd.) W.Y. Zhuang and X.M. Zhang [20,21]. To date, 16 genera and over 100 species of Nectriaceae have been identified in China [22]. These fungi have been isolated from agricultural crops, horticultural plants, and forestry plants distributed across the country [4,9,23,24].

*Cinnamomum camphora* is a common landscaping tree species in China, and research on diseases occurring in these trees has drawn increasing attention. At least 30 diseases associated with *Ci. camphora* trees have been reported in this country, 22 of which are caused by fungal pathogens [14]. Limited research has been conducted on the diversity and pathogenicity of Nectriaceae fungi in *Ci. camphora* trees in China. To date, the only reported disease caused by Nectriaceae fungi affecting these trees is stem cankers caused by *Fusarium decemcellulare* Brick [25]. Recently, during disease surveys on *Eucalyptus* L’Hér. trees in China, we found a leaf spot disease occurring on *Ci. camphora* trees planted in nurseries alongside urban–rural roadways and in urban green areas in Fujian and Guangdong Provinces. The asexual structures of the pathogen present morphological characteristics typical of the Nectriaceae. The aim of this study is to identify these fungi based on multi-gene phylogenetic analyses, and combine the morphological characteristics and evaluate their pathogenicity in *Ci. camphora*.

## 2. Materials and Methods

### 2.1. Disease Symptoms, Sample Collection, and Fungal Isolation

**Figure 1:**
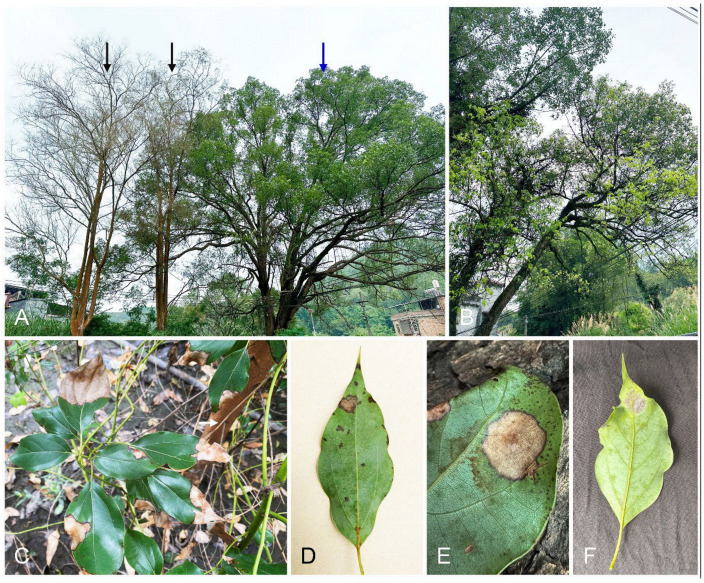
Disease symptoms in *Cinnamomum camphora* trees caused by *Recticladiella inexpectata*. (**A**) One approximately 50-year-old *Ci. camphora* tree (indicated by blue arrow) was planted around two approximately 45-year-old *Eucalyptus exserta* trees (indicated by black arrows). The whole leaves of the *E*. *exserta* trees fell after infection for unknown reasons. New leaves appeared after infection by *R. inexpectata* on the *Ci. camphora* tree. (**B**) Young light-yellow leaves appeared on *Ci. camphora* trees after infection with *R. inexpectata*. (**C**) The infected leaves of a 1-year-old *Ci. camphora* tree were blighted. A *Ci. camphora* tree was planted close to the *E*. *urophylla* hybrid tree (white arrow indicates the stem). (**D**) Water-soaked, small lesions, and light-brown spots on an infected leaf. (**E**) Typical leaf spot on a *Ci. camphora* leaf. (**F**) White conidiophores appeared on the spot of the infected leaf.

In June and July 2024, we conducted surveys of *Eucalyptus* tree diseases in Fujian and Guangdong Provinces in southern China. We found heavy defoliation in *Ci. camphora* trees planted around *Eucalyptus* trees in nurseries, villages, and urban regions (Figure 1A,B). The typical symptoms included leaf blight and spots (Figure 1C). The disease initially presented as water-soaked and gray lesions on the leaves (Figure 1D). As it rapidly spread, the lesions progressed to a dark color and covered a significant portion of the leaf blade (Figure 1E), ultimately leading to defoliation. White conidiophores with the typical morphological characteristics of the Nectriaceae were observed on the spotted leaves (Figure 1F).

Diseased leaves with typical Nectriaceae conidiophores produced on the leaf spots were collected from 31 diseased *Ci. camphora* trees at 13 sites in two provinces: five sites in the Zhangzhou Region, one site in the Longyan Region, one site in the Sanming Region in Fujian Province, five sites in the Heyuan Region, and one site in the Meizhou region in the Guangdong Province. These diseased leaf samples were transported to the laboratory for fungal isolation, morphological study, and further molecular research.

Diseased leaves were incubated in moist dishes (diameter of 70 mm and height of 16 mm; tissue paper moistened with sterile water) at room temperature for 1–2 days to induce Nectriaceae sporulation. Fungal isolates with morphological characteristics typical of the Nectriaceae were isolated from diseased leaves. For single-conidium isolation, each conidiophore mass of Nectriaceae produced on the diseased leaves was transferred to 2% (*v*/*v*) malt extract agar (MEA) (20 g of malt extract powder (Qingdao Hope Bio-Technology Co., Ltd., Qingdao, Shandong, China) and 20 g of agar powder (Beijing Solarbio Science and Technology Co., Ltd., Beijing, China) per liter of water) with a sterile needle under a stereoscopic microscope (Carl Zeiss Suzhou Co., Ltd., Suzhou, Jiangsu, China). After incubation at 25 °C for 3–4 h, the germinated conidia were individually transferred onto fresh MEA under a stereoscopic microscope and incubated at 25 °C for 7–10 days to obtain single-conidium cultures. One single-conidium culture was obtained from each diseased leaf with white conidiophore masses. All obtained single-conidium cultures were maintained in the Culture Collection (CSFF) at the Forest Pathogen Center (FPC), College of Forestry, Fujan Agricultural and Forestry University (FAFU) in Fuzhou, Fujian Province, China. Representative isolates were also deposited at the China General Microbiological Culture Collection Centre (CGMCC), Beijing, China, and dried cultures with sexual and/or asexual structures in a metabolically inactive state served as dried specimens and were deposited in the Mycological Fungarium of the Institute of Microbiology, Chinese Academy of Sciences (HMAS), Beijing, China.

### 2.2. DNA Extraction, PCR Amplification, and Sequencing

Total genomic DNA extraction was performed for all Nectriaceae isolates obtained in this study. Each single-conidium isolate was cultivated on 2% (*w*/*v*) MEA at 25 °C for 7 days. The mycelia were collected using a sterilized bamboo skewer and transferred to 2 mL Eppendorf tubes. The total genomic DNA of each isolate was extracted following the cetyltrimethylammonium bromide (CTAB) method described by Van Burik et al. [26]. The extracted DNA was dissolved by adding 30 μL of TE buffer (1 M Tris–HCl and 0.5 M EDTA, pH 8.0), and 2.5 µL of RNase (10 mg/mL) was added to degrade the RNA. The mixture was incubated at 37 °C for 1 h. The DNA concentration of each isolate was measured by using a NanoDrop Lite Plus spectrophotometer (Thermo Fisher Scientific, Waltham, MA, USA). All DNA samples were diluted to approximately 100 ng/µL by using Water-DEPC Treated Water (Sangon Biotech Co., Ltd., Shanghai, China) and stored at −20 °C for further use.

Based on previous studies, partial gene regions, including translation elongation factor 1-alpha (*tef1*), β-tubulin (*tub2*), calmodulin (*cmdA*), and histone H3 (*his3*), were used as DNA barcodes to distinguish genus and species in Nectriaceae [3,27,28]. Fragments of the *tef1*, *tub2*, *cmdA*, and *his3* genes were amplified by using the primer pairs EF1-728F [27]/EF2 [29], T1 [30]/CYLTUB1R [31], CAL-228F [27]/CAL-2Rd [32], and CYLH3F/CYLH3R [31], respectively. The PCR amplification reaction followed the method described by by Liu et al. [28].

By using the same primers used for PCR amplification, the amplification products of all Nectriaceae isolates were sequenced in the forward and reverse directions. Sequence reactions were performed by Sangon Biotech (Shanghai) Co., Ltd. (Shanghai, China). The *tef1*, *tub2*, *cmdA*, and *his3* gene regions were sequenced for all Nectriaceae isolates obtained in this study. All obtained sequences were edited and assembled by using MEGA v. 7.0 software [33] and submitted to GenBank (https://www.ncbi.nlm.nih.gov; accessed on 6 December 2024).

### 2.3. Multigene Phylogenetic Analyses

By using the sequences of the *tef1*, *tub2*, *cmdA*, and *his3* regions of all isolates obtained in this study, all Nectriaceae isolates were preliminarily identified by conducting a standard nucleotide BLAST search. To conduct phylogenetic analyses, the sequences of the ex-type strains of closely related species were downloaded from the NCBI database (http://www.ncbi.nlm.nih.gov). The sequences generated in the current study and the sequences downloaded from the NCBI database were aligned by using MAFFT online v. 7 (http://mafft.cbrc.jp/alignment/server; accessed on 3 November 2024) [34] with the iterative refinement method (FFT-NS-i setting). The alignments were edited manually with MEGA v. 7.0 software [33] when necessary. The sequences of each of the *tef1*, *tub2*, *cmdA*, and *his3* regions as well as combinations of the four regions, were analyzed.

The maximum likelihood (ML) and Bayesian inference (BI) methods were used for the phylogenetic analyses of the four individual sequences of the *tef1*, *tub2*, *cmdA*, and *his3* regions, as well as for a concatenated dataset of all four regions. The ML analyses were performed by using PHYML v. 3.0 [35]. The default GTR substitution matrix was used for the analyses. In PHYML, the maximum number of retained trees was set to 1000, and nodal support was conducted by performing non-parametric bootstrapping with 1000 replicates. The BI analyses were performed by using MrBayes v. 3.2.6 [36] on the CIPRES Science Gateway v. 3.3. Four Markov chain Monte Carlo (MCMC) chains were run from a random starting tree for generations of five million generations, and the trees were sampled every 100th generation. The first 25% of the trees sampled were discarded as burn-in, and the remaining trees were used to determine the posterior probabilities. The sequence data of *Stachybotrys chartarum* (Ehrenb.) S. Hughes CBS 129.13 were used as an outgroup taxon [3]. The Phylogenetic trees were viewed by using MEGA v. 7.0 [33].

### 2.4. Morphology

Based on the DNA sequence comparisons, the mycelial plugs of all isolates presented as novel species were transferred onto synthetic nutrient-poor agar (SNA) [37] and incubated at 25 °C to test their conidiophore production. The isolates that produced conidiophores on SNA in 14 days were selected for morphological study. Furthermore, autoclaved carnation leaf pieces were added to the SNA cultures to induce greater sporulation [38]. The asexual structures were mounted in sterile water and examined by using a Zeiss Axio Imager. A2 compound microscope (Carl Zeiss Ltd., Jena, Germany).

To induce sexual structures, the isolates of each novel species were crossed with each other in all possible combinations on minimum salt agar (MSA) [39]. Isolates crossed with themselves served as controls. Sterile toothpicks were placed on the surface of MSA media [39]. All crosses and controls were incubated at 25 °C for 4–8 weeks and were considered successful when the isolate combinations or isolates produced perithecia extruding viable ascospores.

For the isolates selected to represent the holotype specimens, 50 measurements were made for each taxonomically informative structure (such as the conidial length and width), and 25 for other structures. For the no-holotype specimens, 25 measurements were made for each taxonomically informative structure and 10 measurements were made for other structures. For the taxonomically informative structures, minimum, maximum, and average (mean) values are presented as (minimum–) (average − standard deviation)–(average + standard deviation) (–maximum). Extremes and averages are presented for other fungal structures.

To determine the optimal growth temperature for novel species, 5 mm mycelial plugs taken from the actively growing edges of the cultures were transferred to fresh MEA plates and incubated at 5–35 °C in 5 °C intervals, with 5 replicate plates per temperature per isolate. Colony diameters were measured after 7 days. Based on 7-day-old cultures grown on MEA at 25 °C, the colony color was described based on the color charts by Rayner [40], and the colony morphology was described. All descriptions were deposited in MycoBank (www.mycobank.org).

### 2.5. Inoculation Tests

The pathogenicity of three isolates of *R*. *inexpectata* (CSFF 26013, CSFF 26015, and CSFF 26033) and two isolates of *Calonectria crousiana* (CSFF 26024 and CSFF 26025) was tested by inoculating these fungi on *Cinnamomum camphora* S.F. Chen, L. Lombard, M.J. Wingf. and X.D. Zhou seedlings. *Cinnamomum camphora* seedlings of 40–60 cm in height with healthy leaves were used for inoculation. Mycelial plugs of 5 mm in diameter were cut from the actively growing margins of the 7-day-old MEA cultures and used to inoculate the leaves of *Ci. camphora* seedlings. For each isolate, 10 mycelial plugs were used to inoculate the abaxial surface of 10 leaves of 3–4 *Ci. camphora* seedlings. For the negative control, 10 sterile MEA plugs were used to inoculate 10 leaves. No wounds were made on the inoculated leaves. All inoculated and negative control seedlings were kept in moist plastic chambers and maintained under stable climatic conditions as follows: 85–99% humidity and 25–27 °C. The entire experiment was repeated a second time using the same method. Inoculation was performed from October to November 2024 at the Forestry Pathogen Center (FPC), College of Forestry, Fujian Agricultural and Forestry University, Fuzhou, Fujian Province, China.

On the day on which the most pathogenic isolate nearly rotted the entire inoculated leaf, the plastic chambers were removed, and all inoculated and negative control leaves were collected. To measure the lesion lengths on each leaf, the diameter of each lesion was measured, and the average diameter (length) of each leaf was calculated. Re-isolation was conducted to confirm Koch’s postulates. Small pieces of the discolored leaves (approximately 0.25 mm × 0.25 mm) were cut from the lesion edge and placed on 2% MEA at room temperature. Re-isolation was performed for all leaves inoculated with each isolate and the negative control. The identities of the re-isolated fungi were verified according to the culture morphology, the fruiting structure morphology, and the disease symptoms produced on the inoculated leaves compared with the original fungi used for inoculation.

The results were analyzed in EXCEL (2019). Single-factor analysis of variance (ANOVA) was performed to define the effects of the fungal isolates on lesion length. To test the significance of the means, *F*-values with *p* < 0.05 were considered significantly different. The standard errors of the means of lesion length for each fungal isolate and the negative control were calculated.

## 3. Results

### 3.1. Fungal Isolation

Conidiophore masses were produced on the diseased leaves of all 31 sampled *Ci. camphora* trees. One or two single-conidium cultures obtained from each sampled tree were selected, and 54 Nectriaceae isolates were used for further study (Table 1) and separated into two groups by conidiophore and conidium morphology. Two isolates (Group One) produced significantly larger conidiophores and conidia than the other 52 isolates (Group Two), and the length of the conidia of the isolates in Group One was nearly twice that of the isolates in Group Two.

### 3.2. Multigene Phylogenetic Analyses

The amplicons generated for the *tef1*, *tub2*, *cmdA*, and *his3* regions of fungi isolated in this study were approximately 480, 570, 685, and 450 bp, respectively. A BLAST search using the sequences of the *tef1*, *tub2*, *cmdA*, and *his3* regions allowed us to divide the 54 isolates obtained in this study into two groups. Two isolates (CSFF 26024 and CSFF 26025), Group One, were of the genus *Calonectria* and were closest to the isolates of Ca. *crousiana* in the Ca. *reteaudii* species complex. The other 52 isolates in Group Two were closest to *Curvicladiella cignea* (Decock and Crous) Decock and Crous based on the *tef1*, *tub2*, and *cmdA* regions and closest to *Calonectria* species based on the *his3* region. The sequences of the ex-type specimen strains and additional strains of all molecularly identified *Calonectria* species in the Ca. *reteaudii* (Bugnic.) C. Booth species complex and the representative *Calonectria* species in each of the other nine species complexes were downloaded from GenBank [28]. The ex-type specimen strains and additional strains of all molecularly identified species of *Curvicladiella* Decock and Crous and the species that were phylogenetically close to *Curvicladiella* were also downloaded from GenBank (Table 2). All downloaded sequences were used for comparisons and phylogenetic analyses.

Isolates CSFF 26024 and CSFF 26025 in Group One were used for phylogenetic analyses. For the 52 isolates in Group Two, the genotype of each isolate was generated based on the *tef1*, *tub2*, *cmdA*, and *his3* regions. Six genotypes were generated, and two isolates of each genotype were used for phylogenetic analyses. A total of 14 isolates were selected.

**Figure 2:**
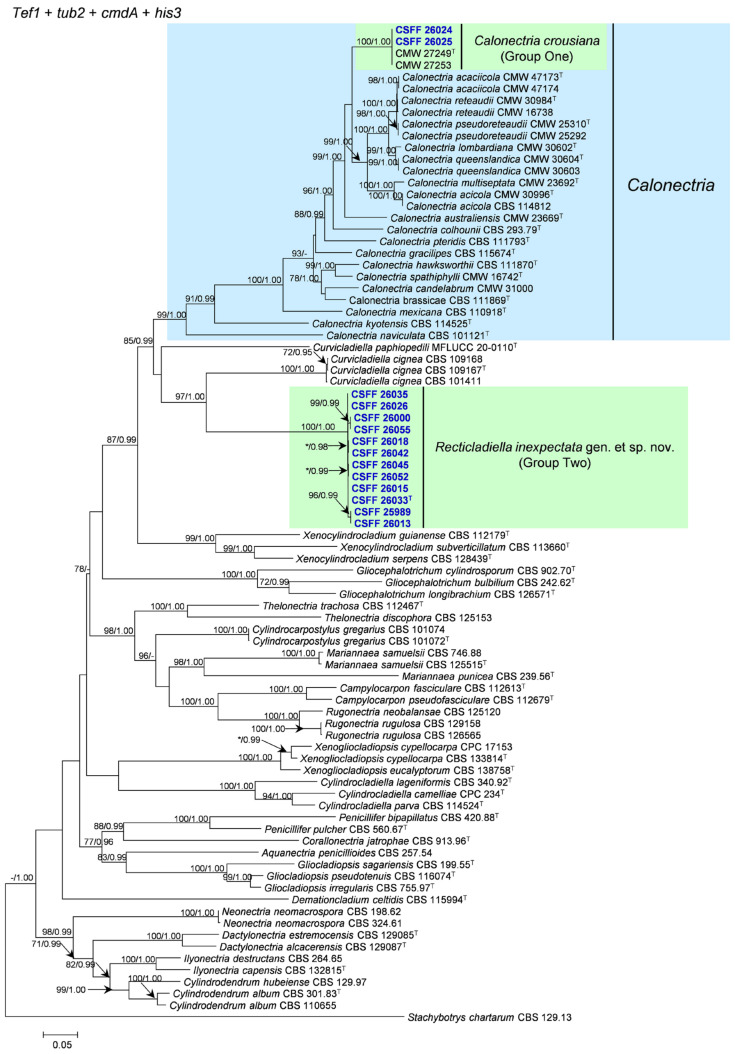
The phylogenetic tree of Nectriaceae species based on maximum likelihood (ML) analyses of the combined *tef1*, *tub2*, *cmdA*, and *his3* gene sequence dataset in this study. Bootstrap values ≥ 70% from the ML analysis and posterior probabilities values _≥ 0.95 obtained from Bayesian inference (BI) are indicated at the nodes as ML/BI. Bootstrap values < 70% or posterior probabilities values < 0.95 are marked with “*”, and absent analysis values are marked with “-”. “*/*”, “*/-”, “-/*”, and “-/-” are not displayed. Ex-type isolates are indicated with “T”. The isolates reported in this study are highlighted in bold and blue.

**Figure 3:**
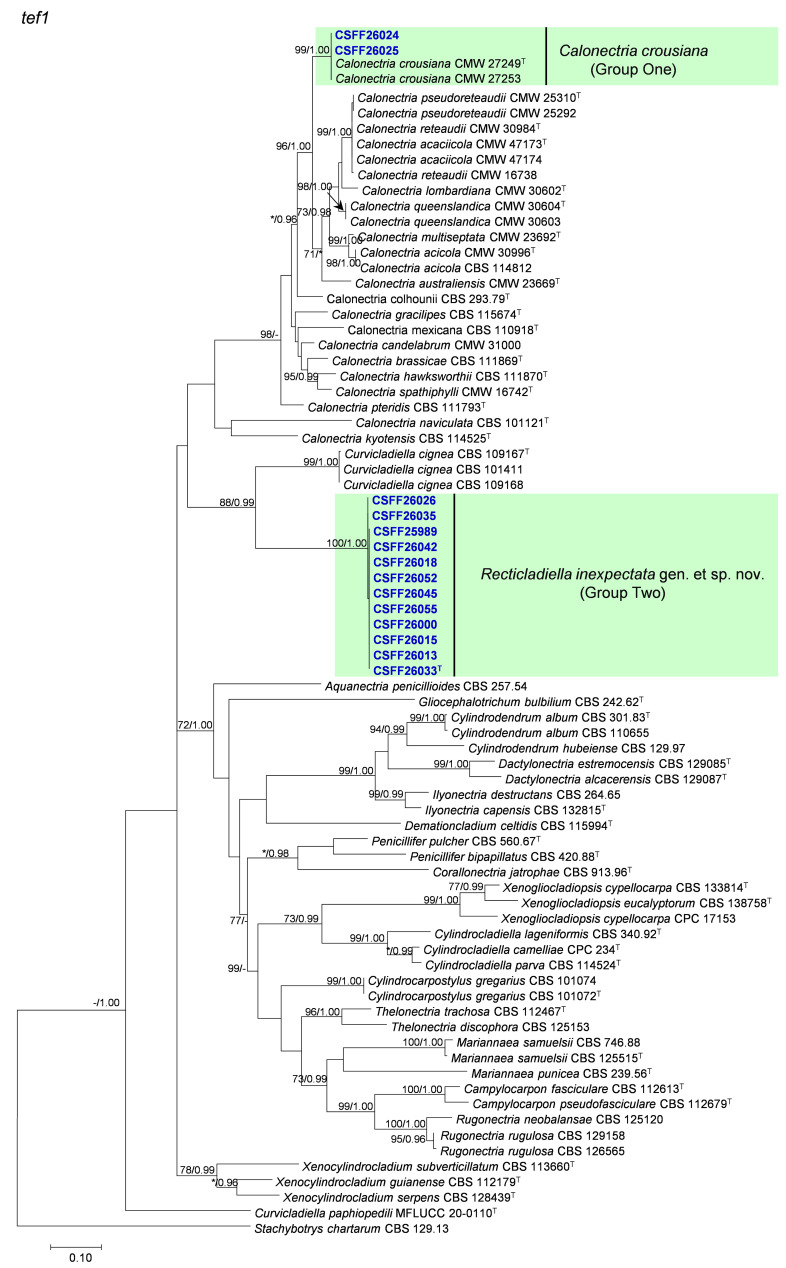
The phylogenetic tree of Nectriaceae species based on maximum likelihood (ML) analyses of the *tef1* gene sequence dataset in this study. Bootstrap values ≥ 70% from the ML analysis and posterior probabilities values _≥ 0.95 obtained from Bayesian inference (BI) are indicated at the nodes as ML/BI. Bootstrap values < 70% or posterior probabilities values < 0.95 are marked with “*”, and absent analysis values are marked with “-”. “*/*”, “*/-”, “-/*”, and “-/-” are not displayed. Ex-type isolates are indicated with “T”. The isolates reported in this study are highlighted in bold and blue.

**Figure 4:**
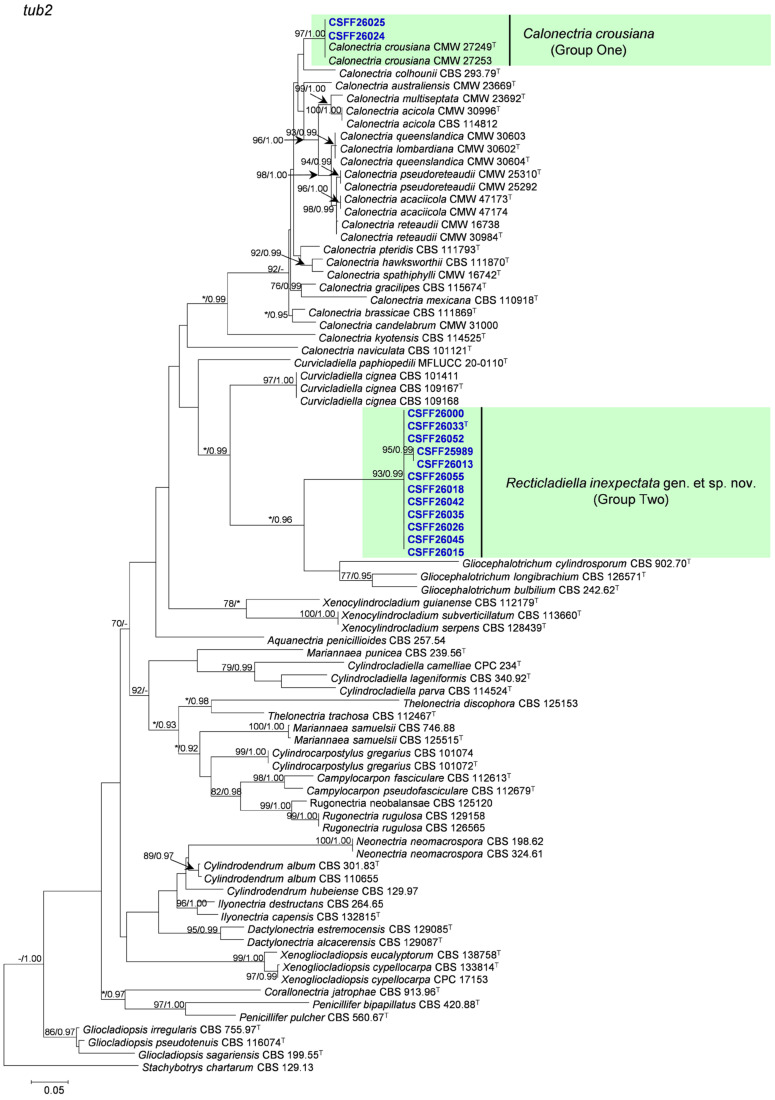
The phylogenetic tree of Nectriaceae species based on maximum likelihood (ML) analyses of the *tub2* gene sequence dataset in this study. Bootstrap values ≥ 70% from the ML analysis and posterior probabilities values _≥ 0.95 obtained from Bayesian inference (BI) are indicated at the nodes as ML/BI. Bootstrap values < 70% or posterior probabilities values < 0.95 are marked with “*”, and absent analysis values are marked with “-”. “*/*”, “*/-”, “-/*”, and “-/-” are not displayed. Ex-type isolates are indicated with “T”. The isolates reported in this study are highlighted in bold and blue.

**Figure 5:**
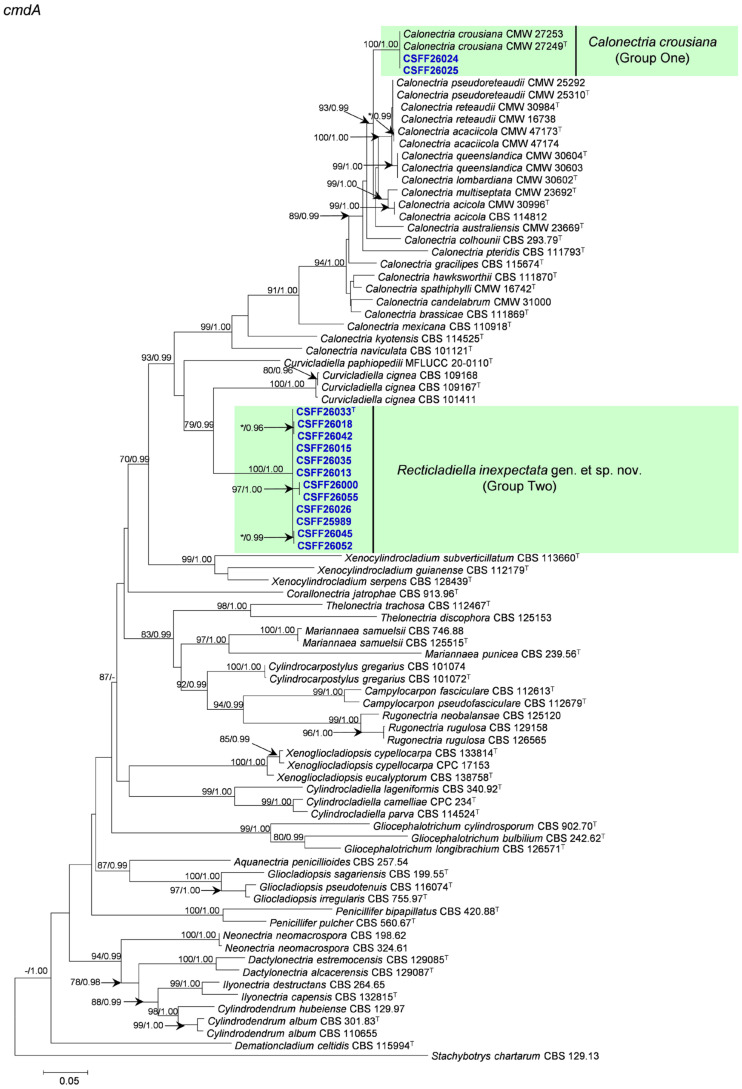
The phylogenetic tree of the Nectriaceae species based on maximum likelihood (ML) analyses of the *cmdA* gene sequence dataset in this study. Bootstrap values ≥ 70% from the ML analysis and posterior probabilities values _≥ 0.95 obtained from Bayesian inference (BI) are indicated at the nodes as ML/BI. Bootstrap values < 70% or posterior probabilities values < 0.95 are marked with “*”, and absent analysis values are marked with “-”. “*/*”, “*/-”, “-/*”, and “-/-” are not displayed. Ex-type isolates are indicated with “T”. The isolates reported in this study are highlighted in bold and blue.

**Figure 6:**
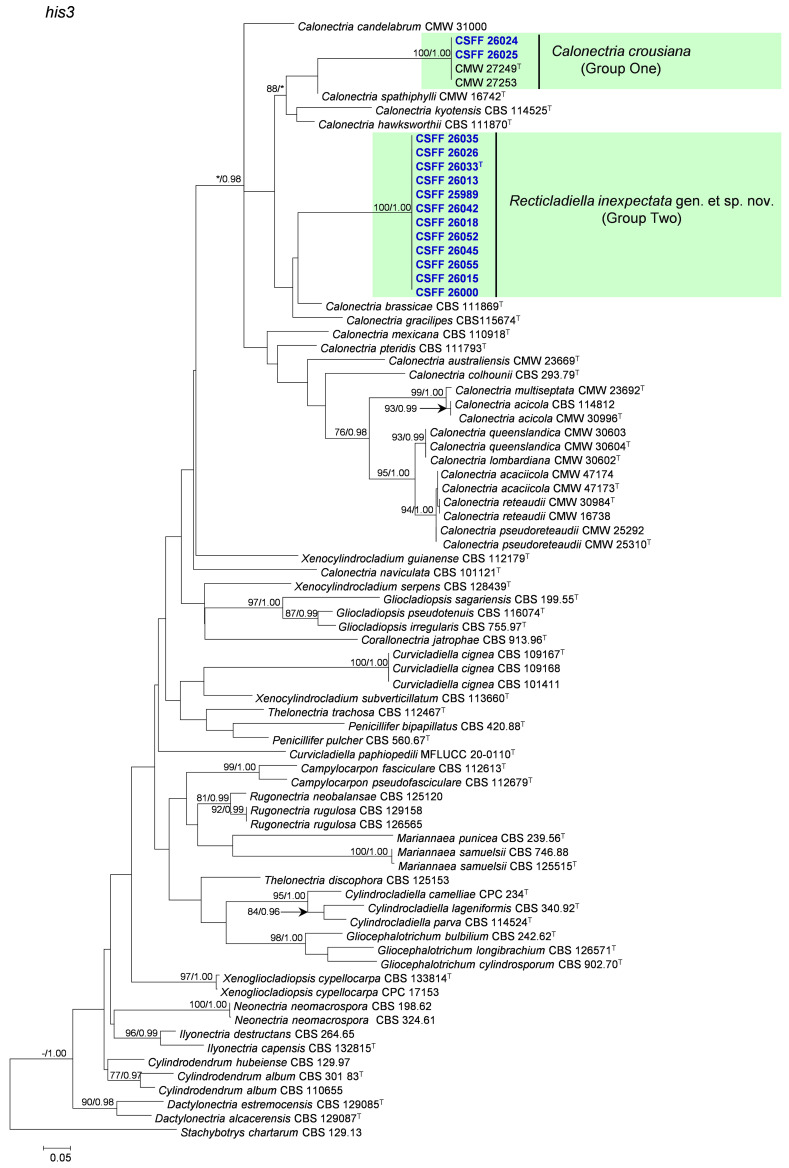
The phylogenetic tree of Nectriaceae species based on maximum likelihood (ML) analyses of the *his3* gene sequence dataset in this study. Bootstrap values ≥ 70% from the ML analysis and posterior probabilities values _≥ 0.95 obtained from Bayesian inference (BI) are indicated at the nodes as ML/BI. Bootstrap values < 70% or posterior probabilities values < 0.95 are marked with “*”, and absent analysis values are marked with “-”. “*/*”, “*/-”, “-/*”, and “-/-” are not displayed. Ex-type isolates are indicated with “T”. The isolates reported in this study are highlighted in bold and blue.

The phylogenetic analysis results indicate that the overall topologies generated from the BI analyses were similar to those from the ML analyses for each of *tef1*, *tub2*, *cmdA*, and *his3* and the four-gene combination datasets. Consequently, only the ML tree with ML bootstrap support values and BI posterior probabilities is presented. The ML tree generated based on a combination of four gene sequences is presented in Figure 2, and those generated based on each of the four gene sequences are presented in Figure 3, Figure 4, Figure 5 and Figure 6. The phylogenetic analyses of *tef1*, *tub2*, *cmdA*, and *his3* and the four-gene combination datasets consistently showed that CSFF 26024 and CSFF 26025 grouped in the same clade with ex-type isolate CMW 27249 of Ca. *crousiana* (Figure 2, Figure 3, Figure 4, Figure 5 and Figure 6). Therefore, isolates CSFF 26024 and CSFF 26025 were identified as Ca. *crousiana*. The 12 isolates in Group Two formed one independent clade for each of the *tef1*, *tub2*, *cmdA*, and *his3* regions, showing a long tree length, high bootstrap and posterior probability support values (100%/1, 93%/0.99, 100%/1, and 100%/1, respectively), and phylogenetic similarity to *Cu*. *cignea* in *tef1* and *cmdA* trees, *Cu*. *cignea* and *Gliocephalotrichum* species in the *tub2* tree, and *Calonectria* species in the *his3* tree (Figure 3, Figure 4, Figure 5 and Figure 6). The combined tree for *tef1*, *tub2*, *cmdA*, and *his3* further demonstrates that the 12 isolates formed one independent clade with long tree length and high bootstrap and posterior probability values (100%/1) which was the most similar to species of *Curvicladiella* and *Calonectria* (Figure 2). Based on the phylogenetic analyses of *tef1*, *tub2*, *cmdA*, and *his3* and their combined datasets, the 12 isolates in Group Two were identified as a novel taxon.

### 3.3. Morphology

Consistent with the phylogenetic analyses, the morphology of the 54 Nectriaceae isolates from *Ci. camphora* was separated into two distinct groups based on the morphological characteristics of the asexual structures, consistent with the two phylogenetic lineages representing the different genera recognized in this study. Both groups produced septate stipe extensions. Isolates in Group One (isolates CSFF 26024, and CSFF 26025) stipe extension tapered toward a clavate vesicle, and macroconidia (1–)3-septate and approximately two times the length of those in Nectriaceae isolates in Group Two, which presented morphological characteristics consistent with Ca. *crousiana* [47]. The stipe extensions of Group Two isolates tapered toward a terminal cell that was straight or occasionally slightly curved, making it morphologically significantly different from the phylogenetically close genera *Calonectria* (stipe extensions tapering toward a terminal vesicle) [47] and *Curvicladiella* (stipe extensions tapering toward a terminal cell that is curved similar to a swan neck) [60].

Based on phylogenetic analyses and morphological characteristics, the Group Two isolates clearly represented a previously undescribed genus and species. Based on three isolates of Group Two (CSFF 26013, CSFF 26015, and CSFF 26033), which represented a novel species, no sexual structures were produced in the crossing tests on MSA. Asexual structures were common for the three isolates on the SNA medium. The unknown genus and species are described as follows:

**Taxonomy**

*Recticladiella* S.F. Chen, *gen. nov.* MycoBank MB 856872.

Etymology: Rectus means straight in Latin, in reference to the terminal cells of the stipe extensions, always straight, which differ from its phylogenetically close genus *Curvicladiella* with curved terminal cells.

Description: Sexual morph not observed. Conidiophores penicillate, hyaline, consisting of a stipe, a penicillate arrangement of fertile branches, and several avesiculate stipe extensions. Stipe septate, hyaline, smooth; stipe extensions septate, straight to flexuous or sinuous, tapering toward a terminal cell; terminal cell straight, occasionally slightly curved. Conidiogenous apparatus with multiple branches. Primary branches aseptate, secondary branches aseptate, and tertiary branches aseptate, with each terminal branch producing multiple phialides. Phialides elongate doliiform to reniform or subcylindrical, hyaline, aseptate, and apex with minute periclinal thickening and inconspicuous collarette. Conidia cylindrical, rounded at both ends, straight, occasionally slightly curved, septate, lacking a visible abscission scar, held in parallel cylindrical clusters by colorless slime.

Type species: *Recticladiella inexpectata* F.Y. Han and S.F. Chen

**Figure 7:**
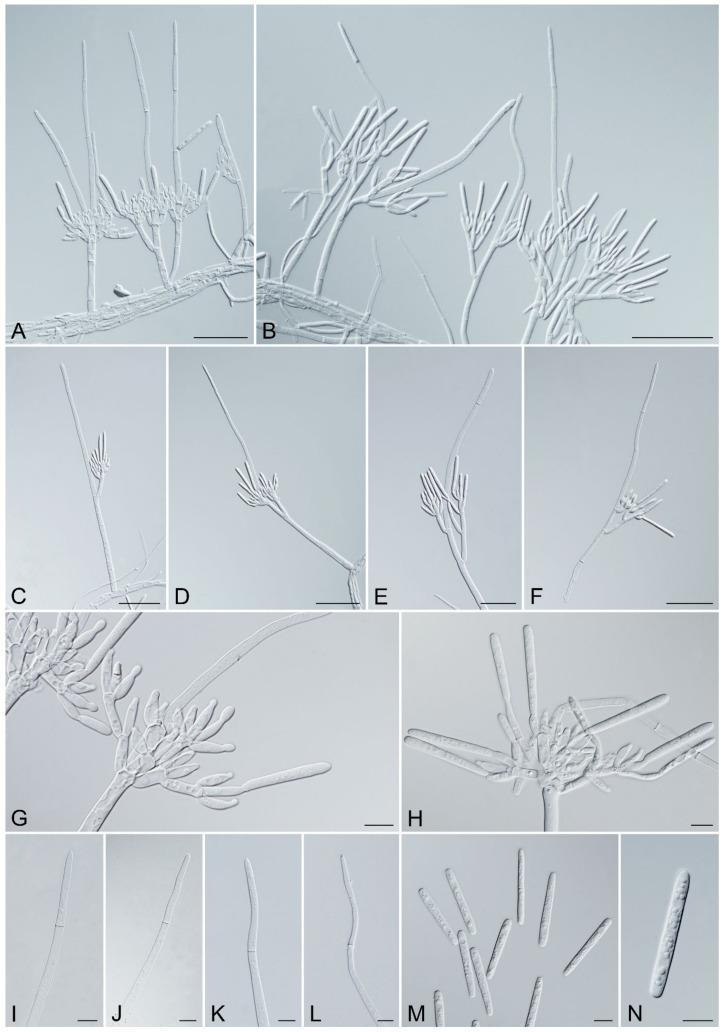
*Recticladiella inexpectata* (ex-type CSFF 26033). (**A**,**B**) Penicillate conidiophores with one to two stipe extensions. (**C**–**F**) Penicillate conidiophores with straight (**C**) to flexuous (**D**–**F**) stipe extensions. (**G**,**H**) Penicillate conidiogenous apparatus. (**I**–**L**) Straight terminal cell (**I**), occasionally slightly curved (**J**–**L**). (**M**,**N**) Conidia. Scale bars: A–F = 50 μm; G–N = 10 μm.

*Recticladiella inexpectata* F.Y. Han and S.F. Chen, sp. nov. MycoBank MB 856873. Figure 7.

Etymology: Inexpectatus means unexpected in Latin, in reference to having identified the new taxon unexpectedly.

Description: Sexual morph not observed. Conidiophores penicillate, hyaline, consisting of a stipe, a penicillate arrangement of fertile branches, and several avesiculate stipe extensions. Stipe septate, up to 3, septate, hyaline, smooth, 33–89.5 × 4–8.5 μm (avg. = 61.5 × 5.5 μm); stipe extensions, 1–2, septate, straight to flexuous or sinuous, 24.5–201.5 μm (avg. = 93.5 μm) long, tapering toward a terminal cell; terminal cell, straight, occasionally slightly curved, 27.5–90 μm (avg. = 49 μm) long, (2–)3–4.5(–5) μm (avg. = 4 μm) in diameter. Conidiogenous apparatus 15–104 μm (avg. = 53 μm) wide, 12–82 μm (avg. = 49.5 μm) long. Primary branches aseptate, 9–34.5 × 3–7 μm (avg. = 20 × 4.5 μm); secondary branches aseptate, 10.5–31.5 × 3–6 μm (avg. = 15 × 4.5 μm); tertiary branches aseptate, 9–19 × 2.5–4.5 μm (avg. = 13 × 4 μm), each terminal branch producing 2–5 phialides. Phialides elongate doliiform to reniform, or subcylindrical, hyaline, aseptate, 7.5–17.5 × 2.5–5 μm (avg. = 12 × 3.5 μm), apex with minute periclinal thickening and inconspicuous collarette. Conidia cylindrical, rounded at both ends, straight, occasionally slightly curved, (32–)36–41(–46.5) × (3.5–)4–5.5(–6) μm (avg. = 38.5 × 5 μm), 1-septate, lacking a visible abscission scar, held in parallel cylindrical clusters by colorless slime.

**Figure 8:**
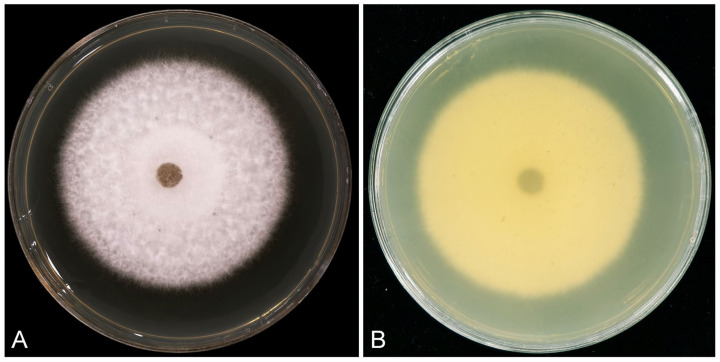
Morphological characteristics of *Recticladiella inexpectata* (ex-type CSFF 26033) colony on MEA after seven days at 25 °C. (**A**) White on the surface. (**B**) Salmon in reverse.

Culture characteristics: Colonies white on the surface and salmon in reverse on MEA after 7 days, with smooth margins, extensive aerial mycelium in the middle and at the margins, and chlamydospores were not observed (Figure 8). Optimal growth temperature at 25 °C, nearly no growth at 5 °C and 35 °C; after 7 days, 5 mm mycelial plug colonies at 5 °C, 10 °C, 15 °C, 20 °C, 25 °C, 30 °C, and 35 °C reached 5.5 mm, 12.3 mm, 21.7 mm, 31.0 mm, 54.6 mm, 52.5 mm, and 5.6 mm, respectively.

Ecology and distribution: *Recticladiella inexpectata* isolated from leaves of 1–150-year-old *Ci. camphora* trees planted in Fujian and Guangdong Provinces, China.

Typus: China, Fujian Province, Zhangzhou Region, Longhai District, Chengxi Town, 24°29′99.5″ N, 117°61′43.19″ E, from the leaf of a *Ci. camphora* tree, July 2024, ShuaiFei Chen, Ying Liu, SuXin Huang, LiSha Wang, and JiaCheng Fang (holotype HMAS 353355, fungarium specimen preserved as dried culture with asexual structures in metabolically inactive state; culture ex-type CSFF 26033 = CGMCC 3.28323). GenBank accession numbers PQ727744 (*tef1*), PQ727798 (*tub2*), PQ727852 (*cmdA*), and PQ727690 (*his3*).

Additional material examined: Fujian Province, Zhangzhou Region, Longhai District, Chengxi Town, 24°33′10.62″ N, 117°64′70.47″ E, from leaf of a *Ci. camphora* tree, July 2024, ShuaiFei Chen, Ying Liu, SuXin Huang, LiSha Wang, and JiaCheng Fang (HMAS 353356, fungarium specimen of dried culture with asexual structures in metabolically inactive state; culture CSFF 26015 = CGMCC 3.28322). GenBank accession numbers PQ727729 (*tef1*), PQ727783 (*tub2*), PQ727837 (*cmdA*), and PQ727675 (*his3*).

Notes: *Recticladiella inexpectata* has been described as a new genus and species based on multi-gene DNA sequence comparisons and morphological characteristics. Before this study, several Nectriaceae genera phylogenetically close to *Recticladiella* produced stipe extensions: *Calonectria*, *Cylindrocladiella* Boesew., *Curvicladiella*, *Gliocephalotrichum* J.J. Ellis and Hesselt., *Xenocylindrocladium* Decock, Hennebert and Crous, and *Xenogliocladiopsis* Crous and W.B. Kendr. *Recticladiella* (stipe extensions single to multiple, septate, no vesicle) can be distinguished from *Calonectria* (stipe extensions: single to multiple, septate, with vesicles), *Cylindrocladiella* (stipe extensions: single, aseptate, with vesicles), *Gliocephalotrichum* (stipe extension number unknown, septate, with vesicles), *Xenocylindrocladium* (stipe extensions: single, septate, no vesicle), and *Xenogliocladiopsis* (stipe extensions: single, septate, no vesicle) based on the morphology of their stipe extensions [28,60,68,77,78,79]. *Recticladiella* is the most morphologically similar to *Curvicladiella*, both of which produce single to multiple stipe extensions that are septate and without a vesicle but with a terminal cell. The stipe extension terminal cells of *Curvicladiella* are curved similar to a swan neck, while those of *Recticladiella* are straight and occasionally slightly curved [60]. Furthermore, *Recticladiella* can be easily distinguished from its most phylogenetically close genus, *Curvicladiella*, based on the DNA sequences of each of the *tef1*, *tub2*, *cmdA*, and *his3* gene regions. *Recticladiella inexpectata* and *Cu*. *cignea* are the type species of their genera. Based on the comparisons between the ex-type isolates of *R*. *inexpectata* (CSFF 26033) and *Cu*. *cignea* (CBS 109167), there are 92, 102, 119, and 80 base differences in the *tef1*, *tub2*, *cmdA*, and *his3* gene regions, respectively.

### 3.4. Inoculation Tests

**Figure 9:**
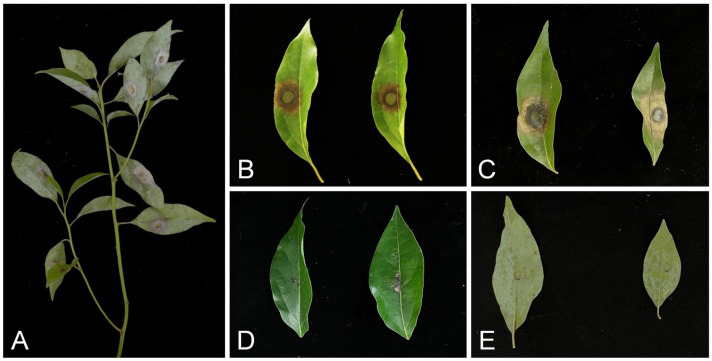
Symptoms on leaves of *Ci. camphora* seedlings caused by *R*. *inexpectata* and Ca. *crousiana* mycelial plugs and MEA plugs (negative controls). (**A**) *Cinnamomum camphora* seedling leaves six days after inoculation with *R*. *inexpectata* isolate CSFF 26015 in experiment two. (**B**,**C**) Leaves six days after inoculation with isolate CSFF 26015 in Experiment One (**B**) and experiment two (**C**). (**D**) Leaves six days after inoculation with Ca. *crousiana* isolate CSFF 26024 in experiment two. (**E**) Leaves six days after inoculation with MEA plugs in Experiment Two.

The inoculation results show that the *R*. *inexpectata* isolates produced lesions on the inoculated leaves in both experiments two days after inoculation. Five days after inoculation, diseased lesions appeared on the leaves inoculated with Ca. *crousiana*. The most pathogenic isolate, CSFF 26015, nearly rotted the entire inoculated leaf 6 days after inoculation (Figure 8A), and the lesion length was measured for all inoculated isolates. The leaves of *Ci. camphora* seedlings inoculated with *R*. *inexpectata* isolates (CSFF 26013, CSFF 26015, and CSFF 26033) and Ca. *crousiana* isolates (CSFF 26024 and CSFF 26025) displayed spots, lesions, and blight (Figure 9A–D). No disease symptoms were observed in the leaves of the negative control seedlings (Figure 9E). The fungal colony shared the same morphological characteristics as the plants originally inoculated with *R*. *inexpectata* (Figure 8) and Ca. *crousiana* [47] isolates that were successfully re-isolated from the leaf lesions. No *Recticladiella* or *Calonectria* were re-isolated from the negative control leaves. Consequently, Koch’s postulates were fulfilled.

**Figure 10:**
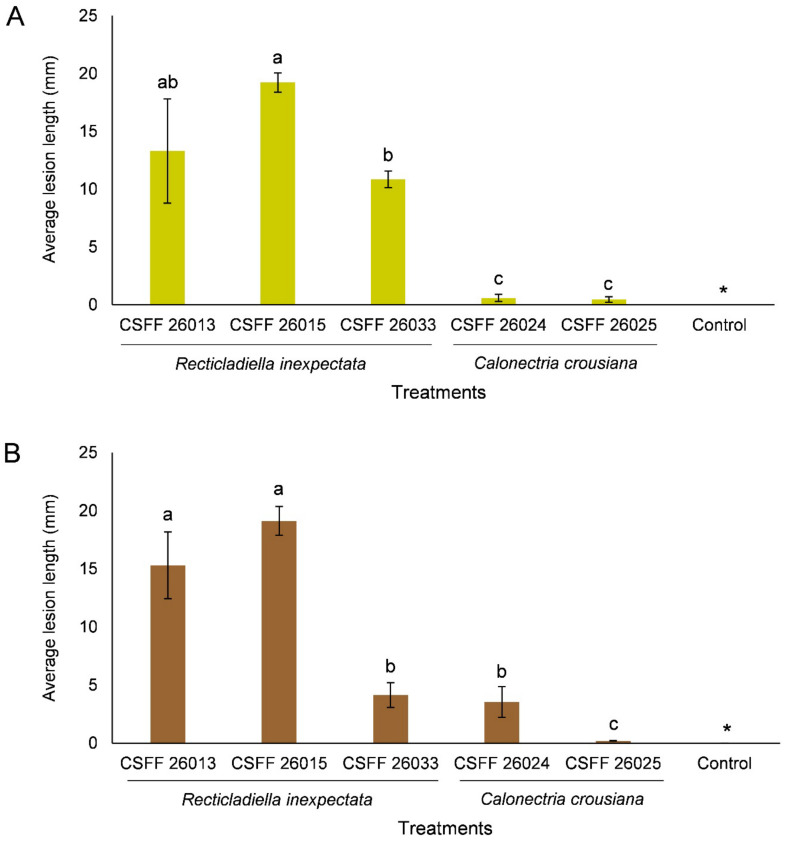
Histograms showing the average lesion lengths on leaves resulting from the inoculation trials of *Ci. camphora* with three isolates of *R*. *inexpectata*, two isolates of Ca. *crousiana*, and a negative control. Two experiments (**A**,**B**) were conducted. The vertical bars represent the standard errors of the means. Different letters above the error bars indicate treatments that were significantly different (*p* = 0.05). “*” represents no lesions produced by the negative control in both experiments.

The ANOVA showed that the results of the replicated inoculation tests were significantly different (*p* < 0.05). Thus, the data for each experiment were analyzed separately. The inoculation results of the two experiments consistently showed that the *R*. *inexpectata* isolates (CSFF 26013, CSFF 26015, and CSFF 26033) were more pathogenic than the Ca. *crousiana* isolates (CSFF 26024 and CSFF 26025) (Figure 10A,B). *Recticladiella inexpectata* isolates CSFF 26013 and CSFF 26015 produced significantly longer lesions than Ca. *crousiana* isolates CSFF 26024 and CSFF 26025. Of the *R*. *inexpectata* isolates, CSFF 26015 produced significantly longer lesions than CSFF 26033 in both experiments (Figure 10A,B).

## 4. Discussion

*Cinnamomum camphora* trees are widely planted in China, especially for landscaping purposes. Research on diseases associated with these trees in China is relatively limited. One potential reason is that people believe that the oil of this tree species can prevent pathogen infection. In this study, we identified two Nectriaceae species, Ca. *crousiana*, and *R*. *inexpectata*, from the diseased leaves of *Ci. camphora* trees planted in southern China. The identification of these two species was supported by four-gene DNA sequence comparisons and morphological characteristics. The inoculation study demonstrated that both species were pathogenetic to the tested *Ci. camphora* seedlings.

*Recticladiella* represents a new genus in the Nectriaceae. It can be distinguished from all other genera in the family based on the DNA sequence data of the *tef1*, *tub2*, c*mdA*, and *his3* gene regions. Morphologically, this genus is the most similar to *Curvicladiella* and *Calonectria*. The morphology of this genus differs from all other Nectriaceae genera in terms of the number of stipe extensions, presence or absence of septa, presence or absence of a vesicle, and curved or straight terminal cells [7,28,60].

Inoculation tests showed that *R*. *inexpectata* caused disease in native *Ci. camphora* seedlings. The *R*. *inexpectata* isolates produced disease symptoms on inoculated *Ci. camphora* leaves within two days, these fungi are clearly leaf pathogens of *Ci. camphora*. *Cinnamomum camphora* was isolated from native *Ci. camphora* trees planted in a range of geographic regions in southern China. *Recticladiella inexpectata* may be a native pathogen in China, and it has the potential to spread to new hosts and areas. We hypothesize that there are more *Recticladiella* species distributed on native *Ci. camphora* trees and other tree species in China.

*Calonectria crousiana* was first isolated and identified from *Eucalyptus* leaves in Fujian Province in 2011 [47]. In this study, Ca. *crousiana* was isolated from one diseased *Ci. camphora* tree planted in one nursery. Inoculation tests in previous studies and this study showed that Ca. *crousiana* is pathogenic to the tested *Eucalyptus* genotypes and *Ci. camphora* seedlings. These results suggest that Ca. *crousiana* may have a wider plant host range. *Calonectria crousiana* may be native to native Chinese *Ci. camphora* trees and spread to non-native *Eucalyptus* trees, but confirmation of this hypothesis requires more systematic research.

Reports of Nectriaceae associated with *Ci. camphora* are currently limited. Few species in four genera of Nectriaceae were reported in *Ci. camphora* (https://fungi.ars.usda.gov; search on 7 December 2024). These include *Calonectria ilicicola* Boedijn and Reitsma, *Cylindrocladiella* sp., *Fusarium oxysporum* Schltdl., and *F. solani* (Mart.) Sacc. in the United States; *F. decemcellulare* Brick in China; and *Neonectria castaneicola* (W. Yamam. and Oyasu) Tak. Kobay. and Hirooka and *N. cinnamomea* (Brayford and Samuels) Brayford and Samuels in Japan [7,25,80,81,82,83,84]. *Fusarium decemcellulare* was proved to cause root rot in *Ci. camphora* seedlings in China [25]. Whether there are other species pathogenic to *Ci. camphora* is still not clear. However, some species were isolated from the diseased tissues of *Ci. camphora*; for instance, *N. castaneicola* was isolated from the cracked rough back of *Ci. camphora* trees [81].

The results of this study expand our understanding of the species diversity, geographic distribution, and pathogenicity of Nectriaceae species in native *Ci. camphora* trees in China. Besides the identification of the new genus and species *R*. *inexpectata*, this is also the first report of Ca. *crousiana* in *Ci. camphora*. These results emphasize that there are many pathogens of woody plants, including some native trees, that remain to be discovered in China. In the future, disease surveys and investigations need to be conducted in wider regions on native *Ci. camphora* trees and plantation trees, such as commercial *Eucalyptus*, to further understand species diversity, pathogenicity, and potential plant health threats in China.

## Figures and Tables

**Table 1:** Isolates sequenced and used for phylogenetic analyses, morphological studies and inoculation tests in this study.

Identity	Genotype ^1^	Isolates No. ^2^	Sample No.	Host/Substrate	Collectors	Location	Coordinates	GenBank Accession No. ^3^			
								*tef1*	*tub2*	*cmdA*	*his3*
*Recticladiella inexpectata*	AAAA	CSFF25976	20240622-5-(1)	*Cinnamomum camphora* leaf	S. F. Chen, Y. Liu, H. B. Dong, L. S. Wang, X. M. Liu	Zhongba Town, Zijin County, Heyuan Region, Guangdong Province	23°42′26.4276″ N, 115°22′0.7428″ E	PQ727708	PQ727762	PQ727816	PQ727654
*R. inexpectata*	AAAA	CSFF25977	20240622-5-(1)	*Ci. camphora* leaf	S. F. Chen, Y. Liu, H. B. Dong, L. S. Wang, X. M. Liu	Zhongba Town, Zijin County, Heyuan Region, Guangdong Province	23°42′26.4276″ N, 115°22′0.7428″ E	PQ727709	PQ727763	PQ727817	PQ727655
*R. inexpectata*	ABAA	CSFF25980	20240622-8-(1)	*Ci. camphora* leaf	S. F. Chen, Y. Liu, H. B. Dong, L. S. Wang, X. M. Liu	Zhongba Town, Zijin County, Heyuan Region, Guangdong Province	23°42′46.7964″ N, 115°22′57.1008″ E	PQ727710	PQ727764	PQ727818	PQ727656
*R. inexpectata*	ABAA	CSFF25984	20240622-8-(3)	*Ci. camphora* leaf	S. F. Chen, Y. Liu, H. B. Dong, L. S. Wang, X. M. Liu	Zhongba Town, Zijin County, Heyuan Region, Guangdong Province	23°42′46.7964″ N, 115°22′57.1008″ E	PQ727711	PQ727765	PQ727819	PQ727657
*R. inexpectata*	ABAA	CSFF25985	20240622-8-(3)	*Ci. camphora* leaf	S. F. Chen, Y. Liu, H. B. Dong, L. S. Wang, X. M. Liu	Zhongba Town, Zijin County, Heyuan Region, Guangdong Province	23°42′46.7964″ N, 115°22′57.1008″ E	PQ727712	PQ727766	PQ727820	PQ727658
*R. inexpectata*	ABAA	CSFF25986	20240622-9-(1)	*Ci. camphora* leaf	S. F. Chen, Y. Liu, H. B. Dong, L. S. Wang, X. M. Liu	Zhongba Town, Zijin County, Heyuan Region, Guangdong Province	23°42′43.5852″ N, 115°22′39.0324″ E	PQ727713	PQ727767	PQ727821	PQ727659
*R. inexpectata*	ABAA	CSFF25987	20240622-9-(1)	*Ci. camphora* leaf	S. F. Chen, Y. Liu, H. B. Dong, L. S. Wang, X. M. Liu	Zhongba Town, Zijin County, Heyuan Region, Guangdong Province	23°42′43.5852″ N, 115°22′39.0324″ E	PQ727714	PQ727768	PQ727822	PQ727660
*R. inexpectata*	AAAA	CSFF25988	20240622-10-(1)	*Ci. camphora* leaf	S. F. Chen, Y. Liu, H. B. Dong, L. S. Wang, X. M. Liu	Zhongba Town, Zijin County, Heyuan Region, Guangdong Province	23°41′45.96″ N, 115°20′57.678″ E	PQ727715	PQ727769	PQ727823	PQ727661
*R. inexpectata*	ABAA	CSFF25989 ^4^	20240622-10-(1)	*Ci. camphora* leaf	S. F. Chen, Y. Liu, H. B. Dong, L. S. Wang, X. M. Liu	Zhongba Town, Zijin County, Heyuan Region, Guangdong Province	23°41′45.96″ N, 115°20′57.678″ E	PQ727716	PQ727770	PQ727824	PQ727662
*R. inexpectata*	AAAA	CSFF25990	20240623-1-(1)	*Ci. camphora* leaf	S. F. Chen, Y. Liu, H. B. Dong, L. S. Wang, X. M. Liu	Zicheng Town, Zijin County, Heyuan Region, Guangdong Province	23°36′32.6196″ N, 115°12′1.7856″ E	PQ727717	PQ727771	PQ727825	PQ727663
*R. inexpectata*	AAAA	CSFF25991	20240623-1-(1)	*Ci. camphora* leaf	S. F. Chen, Y. Liu, H. B. Dong, L. S. Wang, X. M. Liu	Zicheng Town, Zijin County, Heyuan Region, Guangdong Province	23°36′32.6196″ N, 115°12′1.7856″ E	PQ727718	PQ727772	PQ727826	PQ727664
*R. inexpectata*	ABAA	CSFF25992	20240623-3-(3)	*Ci. camphora* leaf	S. F. Chen, Y. Liu, H. B. Dong, L. S. Wang, X. M. Liu	Lanfang Town, Jiaoling County, Meizhou Region, Guangdong Province	24°39′11.6388″ N, 116°11′7.4832″ E	PQ727719	PQ727773	PQ727827	PQ727665
*R. inexpectata*	ABAA	CSFF25993	20240623-3-(3)	*Ci. camphora* leaf	S. F. Chen, Y. Liu, H. B. Dong, L. S. Wang, X. M. Liu	Lanfang Town, Jiaoling County, Meizhou Region, Guangdong Province	24°39′11.6388″ N, 116°11′7.4832″ E	PQ727720	PQ727774	PQ727828	PQ727666
*R. inexpectata*	ABAA	CSFF25994	20240623-3-(4)	*Ci. camphora* leaf	S. F. Chen, Y. Liu, H. B. Dong, L. S. Wang, X. M. Liu	Lanfang Town, Jiaoling County, Meizhou Region, Guangdong Province	24°39′11.6388″ N, 116°11′7.4832″ E	PQ727721	PQ727775	PQ727829	PQ727667
*R. inexpectata*	ABAA	CSFF25995	20240623-3-(4)	*Ci. camphora* leaf	S. F. Chen, Y. Liu, H. B. Dong, L. S. Wang, X. M. Liu	Lanfang Town, Jiaoling County, Meizhou Region, Guangdong Province	24°39′11.6388″ N, 116°11′7.4832″ E	PQ727722	PQ727776	PQ727830	PQ727668
*R. inexpectata*	AAAA	CSFF25996	20240623-3-(5)	*Ci. camphora* leaf	S. F. Chen, Y. Liu, H. B. Dong, L. S. Wang, X. M. Liu	Lanfang Town, Jiaoling County, Meizhou Region, Guangdong Province	24°39′11.6388″ N, 116°11′7.4832″ E	PQ727723	PQ727777	PQ727831	PQ727669
*R. inexpectata*	AAAA	CSFF25997	20240623-3-(5)	*Ci. camphora* leaf	S. F. Chen, Y. Liu, H. B. Dong, L. S. Wang, X. M. Liu	Lanfang Town, Jiaoling County, Meizhou Region, Guangdong Province	24°39′11.6388″ N, 116°11′7.4832″ E	PQ727724	PQ727778	PQ727832	PQ727670
*R. inexpectata*	AABA	CSFF26000 ^4^	20240623-4-(2)	*Ci. camphora* leaf	S. F. Chen, Y. Liu, H. B. Dong, L. S. Wang, X. M. Liu	Shisha Town, Shanghang County, Longyan Region, Fujian Province	25°8′57.3792″ N, 116°36′8.4348″ E	PQ727725	PQ727779	PQ727833	PQ727671
*R. inexpectata*	ABAA	CSFF26012	20240708-1-(2)	*Ci. camphora* leaf	S. F. Chen, Y. Liu, S. X. Huang, L. S. Wang, J. C. Fang	Chengxi Town, Longhai District, Zhangzhou Region, Fujian Province	24°33′10.62″ N, 117°64′70.47″ E	PQ727726	PQ727780	PQ727834	PQ727672
*R. inexpectata*	ABAA	CSFF26013 = CGMCC 3.28321 ^4,5,6^	20240708-1-(2)	*Ci. camphora* leaf	S. F. Chen, Y. Liu, S. X. Huang, L. S. Wang, J. C. Fang	Chengxi Town, Longhai District, Zhangzhou Region, Fujian Province	24°33′10.62″ N, 117°64′70.47″ E	PQ727727	PQ727781	PQ727835	PQ727673
*R. inexpectata*	AAAA	CSFF26014	20240708-1-(3)	*Ci. camphora* leaf	S. F. Chen, Y. Liu, S. X. Huang, L. S. Wang, J. C. Fang	Chengxi Town, Longhai District, Zhangzhou Region, Fujian Province	24°33′10.62″ N, 117°64′70.47″ E	PQ727728	PQ727782	PQ727836	PQ727674
*R. inexpectata*	AAAA	CSFF26015 = CGMCC 3.28322 ^4,5,6^	20240708-1-(3)	*Ci. camphora* leaf	S. F. Chen, Y. Liu, S. X. Huang, L. S. Wang, J. C. Fang	Chengxi Town, Longhai District, Zhangzhou Region, Fujian Province	24°33′10.62″ N, 117°64′70.47″ E	PQ727729	PQ727783	PQ727837	PQ727675
*R. inexpectata*	ABDA	CSFF26018 ^4^	20240708-1-(5)	*Ci. camphora* leaf	S. F. Chen, Y. Liu, S. X. Huang, L. S. Wang, J. C. Fang	Chengxi Town, Longhai District, Zhangzhou Region, Fujian Province	24°33′10.62″ N, 117°64′70.47″ E	PQ727730	PQ727784	PQ727838	PQ727676
*R. inexpectata*	AABA	CSFF26020	20240708-1-(6)	*Ci. camphora* leaf	S. F. Chen, Y. Liu, S. X. Huang, L. S. Wang, J. C. Fang	Chengxi Town, Longhai District, Zhangzhou Region, Fujian Province	24°33′10.62″ N, 117°64′70.47″ E	PQ727731	PQ727785	PQ727839	PQ727677
*R. inexpectata*	AAAA	CSFF26021	20240708-1-(6)	*Ci. camphora* leaf	S. F. Chen, Y. Liu, S. X. Huang, L. S. Wang, J. C. Fang	Chengxi Town, Longhai District, Zhangzhou Region, Fujian Province	24°33′10.62″ N, 117°64′70.47″ E	PQ727732	PQ727786	PQ727840	PQ727678
*R. inexpectata*	AAAA	CSFF26022	20240708-3-(1)	*Ci. camphora* leaf	S. F. Chen, Y. Liu, S. X. Huang, L. S. Wang, J. C. Fang	Chengxi Town, Longhai District, Zhangzhou Region, Fujian Province	24°29′99.51″ N, 117°61′43.19″ E	PQ727733	PQ727787	PQ727841	PQ727679
*R. inexpectata*	AAAA	CSFF26023	20240708-3-(1)	*Ci. camphora* leaf	S. F. Chen, Y. Liu, S. X. Huang, L. S. Wang, J. C. Fang	Chengxi Town, Longhai District, Zhangzhou Region, Fujian Province	24°29′99.51″ N, 117°61′43.19″ E	PQ727734	PQ727788	PQ727842	PQ727680
*R. inexpectata*	BAAA	CSFF26026 ^4^	20240708-3-(3)	*Ci. camphora* leaf	S. F. Chen, Y. Liu, S. X. Huang, L. S. Wang, J. C. Fang	Chengxi Town, Longhai District, Zhangzhou Region, Fujian Province	24°29′99.51″ N, 117°61′43.19″ E	PQ727737	PQ727791	PQ727845	PQ727683
*R. inexpectata*	BAAA	CSFF26027	20240708-3-(3)	*Ci. camphora* leaf	S. F. Chen, Y. Liu, S. X. Huang, L. S. Wang, J. C. Fang	Chengxi Town, Longhai District, Zhangzhou Region, Fujian Province	24°29′99.51″ N, 117°61′43.19″ E	PQ727738	PQ727792	PQ727846	PQ727684
*R. inexpectata*	AAAA	CSFF26028	20240708-3-(4)	*Ci. camphora* leaf	S. F. Chen, Y. Liu, S. X. Huang, L. S. Wang, J. C. Fang	Chengxi Town, Longhai District, Zhangzhou Region, Fujian Province	24°29′99.51″ N, 117°61′43.19″ E	PQ727739	PQ727793	PQ727847	PQ727685
*R. inexpectata*	AAAA	CSFF26029	20240708-3-(4)	*Ci. camphora* leaf	S. F. Chen, Y. Liu, S. X. Huang, L. S. Wang, J. C. Fang	Chengxi Town, Longhai District, Zhangzhou Region, Fujian Province	24°29′99.51″ N, 117°61′43.19″ E	PQ727740	PQ727794	PQ727848	PQ727686
*R. inexpectata*	AAAA	CSFF26030	20240708-3-(5)	*Ci. camphora* leaf	S. F. Chen, Y. Liu, S. X. Huang, L. S. Wang, J. C. Fang	Chengxi Town, Longhai District, Zhangzhou Region, Fujian Province	24°29′99.51″ N, 117°61′43.19″ E	PQ727741	PQ727795	PQ727849	PQ727687
*R. inexpectata*	AAAA	CSFF26031	20240708-3-(5)	*Ci. camphora* leaf	S. F. Chen, Y. Liu, S. X. Huang, L. S. Wang, J. C. Fang	Chengxi Town, Longhai District, Zhangzhou Region, Fujian Province	24°29′99.51″ N, 117°61′43.19″ E	PQ727742	PQ727796	PQ727850	PQ727688
*R. inexpectata*	AAAA	CSFF26032	20240708-3-(6)	*Ci. camphora* leaf	S. F. Chen, Y. Liu, S. X. Huang, L. S. Wang, J. C. Fang	Chengxi Town, Longhai District, Zhangzhou Region, Fujian Province	24°29′99.51″ N, 117°61′43.19″ E	PQ727743	PQ727797	PQ727851	PQ727689
*R. inexpectata*	AAAA	CSFF26033 = CGMCC 3.28323 ^4,5,6,7^	20240708-3-(6)	*Ci. camphora* leaf	S. F. Chen, Y. Liu, S. X. Huang, L. S. Wang, J. C. Fang	Chengxi Town, Longhai District, Zhangzhou Region, Fujian Province	24°29′99.51″ N, 117°61′43.19″ E	PQ727744	PQ727798	PQ727852	PQ727690
*R. inexpectata*	BAAA	CSFF26034	20240708-3-(7)	*Ci. camphora* leaf	S. F. Chen, Y. Liu, S. X. Huang, L. S. Wang, J. C. Fang	Chengxi Town, Longhai District, Zhangzhou Region, Fujian Province	24°29′99.51″ N, 117°61′43.19″ E	PQ727745	PQ727799	PQ727853	PQ727691
*R. inexpectata*	BAAA	CSFF26035 ^4^	20240708-3-(7)	*Ci. camphora* leaf	S. F. Chen, Y. Liu, S. X. Huang, L. S. Wang, J. C. Fang	Chengxi Town, Longhai District, Zhangzhou Region, Fujian Province	24°29′99.51″ N, 117°61′43.19″ E	PQ727746	PQ727800	PQ727854	PQ727692
*R. inexpectata*	AAAA	CSFF26036	20240708-3-(8)	*Ci. camphora* leaf	S. F. Chen, Y. Liu, S. X. Huang, L. S. Wang, J. C. Fang	Chengxi Town, Longhai District, Zhangzhou Region, Fujian Province	24°29′99.51″ N, 117°61′43.19″ E	PQ727747	PQ727801	PQ727855	PQ727693
*R. inexpectata*	AAAA	CSFF26037	20240708-3-(8)	*Ci. camphora* leaf	S. F. Chen, Y. Liu, S. X. Huang, L. S. Wang, J. C. Fang	Chengxi Town, Longhai District, Zhangzhou Region, Fujian Province	24°29′99.51″ N, 117°61′43.19″ E	PQ727748	PQ727802	PQ727856	PQ727694
*R. inexpectata*	ABAA	CSFF26038	20240708-3-(9)	*Ci. camphora* leaf	S. F. Chen, Y. Liu, S. X. Huang, L. S. Wang, J. C. Fang	Chengxi Town, Longhai District, Zhangzhou Region, Fujian Province	24°29′99.51″ N, 117°61′43.19″ E	PQ727749	PQ727803	PQ727857	PQ727695
*R. inexpectata*	AAAA	CSFF26039	20240708-3-(9)	*Ci. camphora* leaf	S. F. Chen, Y. Liu, S. X. Huang, L. S. Wang, J. C. Fang	Chengxi Town, Longhai District, Zhangzhou Region, Fujian Province	24°29′99.51″ N, 117°61′43.19″ E	PQ727750	PQ727804	PQ727858	PQ727696
*R. inexpectata*	AAAA	CSFF26040	20240708-5-(1)	leaf of moren than 150 years’ *Ci. camphora*	S. F. Chen, Y. Liu, S. X. Huang, L. S. Wang, J. C. Fang	Chengxi Town, Longhai District, Zhangzhou Region, Fujian Province	24°27′94.40″ N, 117°59′88.15″ E	PQ727751	PQ727805	PQ727859	PQ727697
*R. inexpectata*	AADA	CSFF26042 ^4^	20240708-8-(4)	*Ci. camphora* leaf	S. F. Chen, Y. Liu, S. X. Huang, L. S. Wang, J. C. Fang	Changqiao Town, Zhangpu County, Zhangzhou Region, Fujian Province	24°19′25.64″ N, 117°58′22.48″ E	PQ727752	PQ727806	PQ727860	PQ727698
*R. inexpectata*	AADA	CSFF26043	20240708-8-(4)	*Ci. camphora* leaf	S. F. Chen, Y. Liu, S. X. Huang, L. S. Wang, J. C. Fang	Changqiao Town, Zhangpu County, Zhangzhou Region, Fujian Province	24°19′25.64″ N, 117°58′22.48″ E	PQ727753	PQ727807	PQ727861	PQ727699
*R. inexpectata*	AACA	CSFF26044	20240708-12-(1)	*Ci. camphora* leaf	S. F. Chen, Y. Liu, S. X. Huang, L. S. Wang, J. C. Fang	Shuyang Town, Nanjing County, Zhangzhou Region, Fujian Province	24°64′07.52″ N, 117°11′66.80″ E	PQ727754	PQ727808	PQ727862	PQ727700
*R. inexpectata*	AACA	CSFF26045 ^4^	20240708-12-(1)	*Ci. camphora* leaf	S. F. Chen, Y. Liu, S. X. Huang, L. S. Wang, J. C. Fang	Shuyang Town, Nanjing County, Zhangzhou Region, Fujian Province	24°64′07.52″ N, 117°11′66.80″ E	PQ727755	PQ727809	PQ727863	PQ727701
*R. inexpectata*	AABA	CSFF26047	20240708-12-(2)	*Ci. camphora* leaf	S. F. Chen, Y. Liu, S. X. Huang, L. S. Wang, J. C. Fang	Shuyang Town, Nanjing County, Zhangzhou Region, Fujian Province	24°64′07.52″ N, 117°11′66.80″ E	PQ727756	PQ727810	PQ727864	PQ727702
*R. inexpectata*	AABA	CSFF26050	20240708-12-(4)	*Ci. camphora* leaf	S. F. Chen, Y. Liu, S. X. Huang, L. S. Wang, J. C. Fang	Shuyang Town, Nanjing County, Zhangzhou Region, Fujian Province	24°64′07.52″ N, 117°11′66.80″ E	PQ727757	PQ727811	PQ727865	PQ727703
*R. inexpectata*	AACA	CSFF26052 ^4^	20240708-12-(5)	*Ci. camphora* leaf	S. F. Chen, Y. Liu, S. X. Huang, L. S. Wang, J. C. Fang	Shuyang Town, Nanjing County, Zhangzhou Region, Fujian Province	24°64′07.52″ N, 117°11′66.80″ E	PQ727758	PQ727812	PQ727866	PQ727704
*R. inexpectata*	AABA	CSFF26054	20240708-12-(6)	*Ci. camphora* leaf	S. F. Chen, Y. Liu, S. X. Huang, L. S. Wang, J. C. Fang	Shuyang Town, Nanjing County, Zhangzhou Region, Fujian Province	24°64′07.52″ N, 117°11′66.80″ E	PQ727759	PQ727813	PQ727867	PQ727705
*R. inexpectata*	AABA	CSFF26055 ^4^	20240708-12-(6)	*Ci. camphora* leaf	S. F. Chen, Y. Liu, S. X. Huang, L. S. Wang, J. C. Fang	Shuyang Town, Nanjing County, Zhangzhou Region, Fujian Province	24°64′07.52″ N, 117°11′66.80″ E	PQ727760	PQ727814	PQ727868	PQ727706
*R. inexpectata*	ABAA	CSFF26082	20240710-1-(2)	*Ci. camphora* leaf	S. F. Chen, Y. Liu, S. X. Huang, L. S. Wang, J. C. Fang	Suburb of Yongan City, Yongan County, Sanming Region, Fujian Province	25°92′20.84″ N, 117°35′61.07″ E	PQ727761	PQ727815	PQ727869	PQ727707
*Calonectria crousiama*	AAAA	CSFF26024 ^4^	20240708-3-(2)	*Ci. camphora* leaf	S. F. Chen, Y. Liu, S. X. Huang, L. S. Wang, J. C. Fang	Chengxi Town, Longhai District, Zhangzhou Region, Fujian Province	24°29′99.51″ N, 117°61′43.19″ E	PQ727735	PQ727789	PQ727843	PQ727681
Ca. *crousiama*	AAAA	CSFF26025 ^4^	20240708-3-(2)	*Ci. camphora* leaf	S. F. Chen, Y. Liu, S. X. Huang, L. S. Wang, J. C. Fang	Chengxi Town, Longhai District, Zhangzhou Region, Fujian Province	24°29′99.51″ N, 117°61′43.19″ E	PQ727736	PQ727790	PQ727844	PQ727682

^1^ Genotype within each *Recticladiella* and *Calonectria* species, determined by sequences of the *tef1*, *tub2*, *cmdA,* and *his3* regions. ^2^ CSFF: Culture Collection located at Forest Pathogen Center (FPC), College of Forestry, Fujan Agricultural and Forestry University, Fuzhou, Fujian Province, China; CGMCC: China General Microbiological Culture Collection Centre, Beijing, China. ^3^ *tef1* = translation elongation factor 1-alpha; *tub2* = β-tubulin; *cmdA* = calmodulin; *his3* = histone H3. ^4^ Isolates used for phylogenetic analyses. ^5^ Isolates used for morphological studies. ^6^ Isolates used for inoculation tests. ^7^ Ex-type isolate of the species.

**Table 2:** Isolates from other studies used in the phylogenetic analyses for this study.

Species	Isolate No. ^1,2^	Other Collection No.	Substrate	Collector/Depositor	Locality	GenBank Accession No. ^3,4,5^				References of Source of the Isolates/Sequencing Data
						*tef1*	*tub2*	*cmdA*	*his3*	
*Aquanectria penicillioides*	CBS 257.54	ATCC 16261	*Acer* sp.	F.V. Ranzoni	USA	KM231865	KM232000	KM231275	N/A ^4^	[3]
*Calonectria acaciicola*	CMW 47174	CBS 143558	Soil (*Acacia auriculiformis* plantation)	N.Q. Pham and T.Q. Pham	Do Luong, Nghe An, Vietnam	MT412691	MT412931	MT335161	MT335400	[28,41]
*Calonectria acaciicola*	CMW 47173^T^	CBS 143557	Soil (*A. auriculiformis* plantation)	N.Q. Pham and T.Q. Pham	Do Luong, Nghe An, Vietnam	MT412690	MT412930	MT335160	MT335399	[28,41]
*Calonectria acicola*	CBS 114812	CMW 51216	*Phoenix canariensis*	H. Pearson	Northland, New Zealand	MT412693	MT412933	MT335163	MT335402	[28,42,43]
*Calonectria acicola*	CMW 30996^T^		*P. canariensis*	H. Pearson	Northland, New Zealand	MT412692	MT412932	MT335162	MT335401	[28,42,43]
*Calonectria australiensis*	CMW 23669^T^	CBS 112954; CPC 4714	*Ficus pleurocarpa*	C. Pearce and B. Paulus	Queensland, Australia	MT412723	MT412946	MT335192	MT335432	[28,43,44]
*Calonectria brassicae*	CBS 111869^T^	CPC 2409	*Argyreia splendens*	F. Bugnicourt	Indonesia	MT412733	MT412955	MT335202	MT335442	[7,28,43,45]
*Calonectria candelabrum*	CMW 31000	CPC 1675; UFV 117	*Eucalyptus* sp.	A.C. Alfenas	Amazonas, Brazil	MT412738	MT412959	MT335207	MT335447	[3,7,28,43]
*Calonectria colhounii*	CBS 293.79^T^	CMW 30999	*Camellia sinensis*	A. Peerally	Mauritius	GQ267301	DQ190564	GQ267373	DQ190639	[7,28,43,44,46]
*Calonectria crousiana*	CMW 27253	CBS 127199	*Eucalyptus grandis*	M.J. Wingfield	Fujian, China	MT412762	MT412983	MT335231	MT335471	[28,47]
*Calonectria crousiana*	CMW 27249^T^	CBS 127198	*E. grandis*	M.J. Wingfield	Fujian, China	MT412761	MT412982	MT335230	MT335470	[28,47]
*Calonectria gracilipes*	CBS 115674^T^	CMW 51227; STE-U 1153	Soil	M.J. Wingfield	La Selva, Colombia	MT412783	MT413001	MT335252	MT335492	[7,28,44,48]
*Calonectria hawksworthii*	CBS 111870^T^	CMW 51194; CPC 2405	*Nelumbo nucifera*	A. Peerally	Pamplemousses garden, Mauritius	MT412785	MT413003	MT335254	MT335494	[7,28]
*Calonectria kyotensis*	CBS 114525^T^	ATCC 18834; CMW 51824; CPC 2367	*Robinia pseudoacacia*	T. Terashita	Japan	MT412802	MT413019	MT335271	MT335511	[7,28,45,49]
*Calonectria lombardiana*	CMW 30602^T^	CBS 112634; CPC 4233; Lynfield 417	*Xanthorrhoea australis*	T. Baigent	Victoria, Australia	MT412926	MT413133	MT335395	MT335635	[7,28,44,50]
*Calonectria mexicana*	CBS 110918^T^	CMW 9055; STE-U 927	Soil	M.J. Wingfield	Uxmal, Yucatan, Mexico	FJ972526	AF210863	GQ267396	FJ972460	[7,28,43,51,52]
*Calonectria multiseptata*	CMW 23692^T^	CBS 112682; CPC 1589	*E. grandis*	M.J. Wingfield	North Sumatra, Indonesia	MT412830	MT413044	MT335299	MT335539	[7,28,44,53]
*Calonectria naviculata*	CBS 101121^T^	CMW 30974	Leaf litter	R.F. Castaneda	Joao Pessoa, Brazil	GQ267317	GQ267211	GQ267399	GQ267252	[3,43]
*Calonectria pseudoreteaudii*	CMW 25292	CBS 123696	*E. urophylla × E. grandis*	M.J. Wingfield and X.D. Zhou	Guangdong, China	MT412886	MT413097	MT335355	MT335595	[28,50]
*Calonectria pseudoreteaudii*	CMW 25310^T^	CBS 123694	*E. urophylla × E. grandis*	M.J. Wingfield and X.D. Zhou	Guangdong, China	MT412885	MT413096	MT335354	MT335594	[28,50]
*Calonectria pteridis*	CBS 111793^T^	ATCC 34395; CMW 16736; CPC 2372	*Arachniodes adiantiformis*	F. Schickedanz	USA	FJ918563	DQ190578	GQ267413	DQ190679	[7,28,43,44,51,54]
*Calonectria queenslandica*	CMW 30603	CBS 112155; CPC 3210	*E. pellita*	P.Q Thu and K.M. Old	Lannercost, Queensland, Australia	MT412899	MT413109	MT335368	MT335608	[28,50,55]
*Calonectria queenslandica*	CMW 30604^T^	CBS 112146; CPC 3213	*E. urophylla*	B. Brown	Lannercost, Queensland, Australia	MT412898	MT413108	MT335367	MT335607	[28,50,55]
*Calonectria reteaudii*	CMW 30984^T^	CBS 112144; CPC 3201	*E. camaldulensis*	M.J. Dudzinski and P.Q. Thu	Chon Thanh, Binh Phuoc, Vietnam	MT412901	MT413111	MT335370	MT335610	[7,28,44,55]
*Calonectria reteaudii*	CMW 16738	CBS 112143; CPC 3200	*Eucalyptus* sp. (leaves)	M.J. Dudzinski and P.Q. Thu	Binh Phuoc, Vietnam	MT412902	MT413112	MT335371	MT335611	[7,28,44,55]
*Calonectria spathiphylli*	CMW 16742^T^	ATCC 44730; CBS 114540; STE-U 2185	*Spathiphyllum* sp.	C.L. Schoulties	Florida, USA	MT412905	MT413115	MT335374	MT335614	[7,28,43,45,56]
*Campylocarpon* *fasciculare*	CBS 112613^T^	CPC 3970	*Vitis* sp.	F. Halleen	South Africa	F735691	AY677221	KM231297	JF735502	[3,57,58]
*Campylocarpon pseudofasciculare*	CBS 112679^T^	CPC 5472	*Vitis vinifera*	F. Halleen	South Africa	JF735692	AY677214	KM231298	JF735503	[3,57,58]
*Corallonectria jatrophae*	CBS 913.96^T^	GJS 9618	Unknown tree	G.J. Samuels	Puerto Rico	KM231863	KC479787	KM231273	KM231457	[3,59]
*Curvicladiella cignea*	CBS 109167^T^	CPC 1595; MUCL 40269	Leaf litter	C. Decock	French Guiana	KM231867	KM232002	KM231287	KM231461	[3,60]
*Curvicladiella cignea*	CBS 109168	CPC 1594	Decaying seed	C. Decock	French Guiana	KM231868	KM232003	KM231286	KM231460	[3,60]
*Curvicladiella cignea*	CBS 101411		Decaying seed	C. Decock	French Guiana	KM231886	KM232001	KM231285	KM231459	[3,60]
*Curvicladiella paphiopedili*	MFLUCC 20-0110^T^	GZCC 19-0342	*Paphiopedilum* sp.	L.C. Song	Guizhou, China	MT294103	MT294102	MT294104	MT294105	[61]
*Cylindrocladiella camelliae*	CPC 234^T^	PPRI 3990; IMI 346845	*E. grandis*	P.W. Crous	South Africa	JN099087	AY793471	KM231280	AY793509	[3,62,63]
*Cylindrocarpostylus gregarius*	CBS 101072^T^		*Hylurgops palliatus*	R. Kirschner	Germany	KM231870	KM232005	KM231292	N/A	[3,64]
*Cylindrocarpostylus gregarius*	CBS 101074		*Picea abies*	R. Kirschner	Germany	KM231869	KM232004	KM231291	N/A	[3,64]
*Cylindrocladiella lageniformis*	CBS 340.92^T^	PPRI 4449; UFV 115	*Eucalyptus* sp.	A.C. Alfenas	Brazil	JN099003	AY793481	KM231279	AY793520	[62,65]
*Cylindrocladiella parva*	CBS 114524^T^	ATCC 28272; CPC 2370	*Telopea speciosissima*	H.J. Boesewinkel	New Zealand	JN099009	AY793486	KM231281	AY793526	[62,65]
*Cylindrodendrum album*	CBS 110655		Soil	F.X. Prenafeta-Boldú	The Netherland	KM231890	KM232022	KM231323	KM231485	[3]
*Cylindrodendrum album*	CBS 301.83^T^	ATCC 46842; IMI 255534	*Fucus distichus*	R.C. Summerbell	Canada	KM231889	KM232021	KM231322	KM231484	[3]
*Cylindrodendrum hubeiense*	CBS 129.97		*Viscum album*	W. Gams	France	KM231891	KM232023	KM231324	KM231486	[3]
*Dactylonectria alcacerensis*	CBS 129087^T^	CPC 19172	*Vitis vinifera*	A. Cabral and H. Oliveira	Portugal	JF735819	AM419111	KM231330	JF735630	[3,57]
*Dactylonectria estremocensis*	CBS 129085^T^	CPC 19170	*V. vinifera*	C. Rego and T. Nascimento	Portugal	JF735807	JF735448	KM231328	JF735617	[3,57]
*Demationcladium celtidis*	CBS 115994^T^		*Celtis tala*	N. Allegrucci	Argentina	KM231864	N/A	KM231274	N/A	[3,66]
*Gliocephalotrichum bulbilium*	CBS 242.62^T^	ATCC 22228; IFO 9325; IMI 096357; MUCL 18575; NRRL 2899; QM 9007	Soil	L.J. Wickerham	USA	KM231892	DQ377831	KM231283	KF513326	[3,67]
*Gliocephalotrichum cylindrosporum*	CBS 902.70^T^	ATCC 22229; IFO 9326; IMI 155704; MUCL 18576; QM 9009	Soil	C. Klinsukont	Thailand	KF513408 *^5^	DQ377841	KM231284	KF513353	[3,67,68]
*Gliocephalotrichum longibrachium*	CBS 126571^T^	MUCL 46693	Leaf litter	C. Decock and V. Robert	French Guiana	KF513435 *	DQ377835	KM231282	KF513367	[3,67,68]
*Gliocladiopsis irregularis*	CBS 755.97^T^	CPC 718	Soil	A.C. Alfenas	Indonesia	KF513449 *	JQ666133	KM231278	JQ666023	[3,64]
*Gliocladiopsis pseudotenuis*	CBS 116074^T^	CPC 706	Soil	M.J. Wingfield	China	JQ666099 *	JQ666140	KM231277	JQ666030	[3,64]
*Gliocladiopsis sagariensis*	CBS 199.55^T^		Soil	S.B. Saksena	India	JQ666106 *	JQ666141	KM231276	JQ666031	[3,64]
*Ilyonectria capensis*	CBS 132815^T^		*Protea* sp.	K. Bezuidenhout	South Africa	JX231119	JX231103	KM231319	JX231135	[3,69]
*Ilyonectria destructans*	CBS 264.65		*Cyclamen persicum*	L. Nilsson	Sweden	JF735695	AY677256	KM231317	JF735506	[3,57,58]
*Mariannaea punicea*	CBS 239.56^T^		Soil	J. Meyer	Zaire	KM231876	AY624244	KM231299	KM231469	[3,70]
*Mariannaea samuelsii*	CBS 125515^T^	DAOM 235814; KAS 1307	Soil	J. Bissett	Guatemala	KM231883	KM232015	KM231308	KM231478	[3,71]
*Mariannaea samuelsii*	CBS 746.88	CTR 7113	Bark	C.T. Rogerson	Jamaica	KM231882	KM232014	KM231307	KM231477	[3,71]
*Neonectria neomacrospora*	CBS 324.61	DSM 62489	*Abies concolor*	J.A. von Arx	The Netherlands	HM364335 *	DQ789875	KM231313	JF735599	[3,57,72]
*Neonectria neomacrospora*	CBS 198.62	BBA 9628; IMI 113890	*A.s concolor*	W. Gerlach	Germany	JF735788 *	DQ789866	KM231312	KM231481	[3,72]
*Penicillifer bipapillatus*	CBS 420.88^T^		Bark	C.T. Rogerson	Venezuela	KM231860	KM231996	KM231270	KM231454	[3,73]
*Penicillifer pulcher*	CBS 560.67^T^	ATCC 18931; MUCL 11607	Soil	J.H. van Emden	The Netherlands	KM231862	KM231998	KM231272	KM231456	[3,74]
*Rugonectria neobalansae*	CBS 125120	GJS 85219	Dead tree	G.J. Samuels	Indonesia	KM231874	HM352869	KM231294	KM231466	[3,75]
*Rugonectria rugulosa*	CBS 126565	GJS 091245	Dead tree	Y. Hirooka	Venezuela	KM231873	KM232007	KM231296	KM231468	[3,75]
*Rugonectria rugulosa*	CBS 129158		N/A	Y. Hirooka	USA	KM231872	JF832911	KM231295	KM231467	[3,75]
*Stachybotrys chartarum*	CBS 129.13		N/A	H.A. Dale	N/A	KM231994	KM232127	KM231452	KM231588	[3]
*Thelonctria discophora*	CBS 125153	AR 4324	*Piuns radiata*	A.Y. Rossman	New Zealand	KM231897	HM352860	KM231327	KM231489	[3]
*Thelonctria trachosa*	CBS 112467^T^	GJS 9245; IMI 352560	Bark	D. Bradford and G.J. Samuels	Scotland	KM231896	AY677258	KM231326	KM231488	[3,58]
*Xenocylindrocladium guianense*	CBS 112179^T^	CPC 3496; MUCL 41975	Plant litter	C. Decock	French Guiana	KM231895	AF320197	KM231289	KM231463	[3,76]
*Xenocylindrocladium serpens*	CBS 128439^T^	MUCL 39315	Bark	G.L. Hennebert	Ecuador	KM231894	AF320196 *	KM231290	KM231464	[3,77]
*Xenocylindrocladium subverticilatum*	CBS 113660^T^	CPC 3397; MUCL 41834	Plant litter	C. Decock and O. Laurence	Singapore	KM231893	AF320196	KM231288	KM231462	[3,76]
*Xenogliocladiopsis cypellocarpa*	CBS 133814^T^	CPC 19417	*E. cypellocarpa*	P.W. Crous	Australia	KM231885	KM232017	KM231310	KM231479	[3]
*Xenogliocladiopsis cypellocarpa*	CPC 17153		*Eucalyptus* sp.	P.W. Crous	Australia	KM231886	KM232018	KM231311	KM231480	[3]
*Xenogliocladiopsis eucalyptorum*	CBS 138758^T^	CPC 16271	*Eucalyptus* sp.	P.W. Crous	South Africa	KM231884	KM232016	KM231309	N/A	[3,78]

^1^ T: ex-type isolates of the species. ^2^ AR: Amy Y. Rossman working collection; ATCC: American Type Culture Collection, Virginia, USA; CBS: Westerdijk Fungal Biodiversity Institute, Utrecht, The Netherlands; CERC: China Eucalypt Research Centre, Zhanjiang, Guangdong Province, China; CMW: Culture collection of the Forestry and Agricultural Biotechnology Institute (FABI), University of Pretoria, Pretoria, South Africa; CPC: Pedro Crous working collection housed at Westerdijk Fungal Biodiversity Institute; GZCC: Guizhou Culture Collection, Guiyang, Guizhou Province, China; IMI: International Mycological Institute, CABI Bioscience, Egham, Bakeham Lane, UK; MFLUCC: Mae Fah Luang University Culture Collection, Chiang Rai, Thailand; MUCL: Mycotheque, Laboratoire de Mycologie Systematique st Appliqee, I’Universite, Louvian-la-Neuve, Belgium; STE-U: Department of Plant Pathology, University of Stellenbosch, South Africa; –: no other collection number. ^3^ *tef1*: translation elongation factor 1-alpha; *tub2*: β-tubulin; *cmdA*: calmodulin; *his3*: histone H3. ^4^ N/A represents data that is not available. ^5^ * represents data that is confused with other isolate, and the relative data were not used in this study.

## Data Availability

The original contributions presented in the study are included in the article, further inquiries can be directed to the corresponding author.
